# Comparative Transcriptome Analysis Reveals Molecular Defensive Mechanism of *Arachis hypogaea* in Response to Salt Stress

**DOI:** 10.1155/2020/6524093

**Published:** 2020-02-26

**Authors:** Hao Zhang, Xiaobo Zhao, Quanxi Sun, Caixia Yan, Juan Wang, Cuiling Yuan, Chunjuan Li, Shihua Shan, Fengzhen Liu

**Affiliations:** ^1^State Key Laboratory of Crop Biology and College of Agronomy, Shandong Agricultural University, Tai'an, Shandong 271018, China; ^2^Shandong Peanut Research Institute, Qingdao, Shandong 266000, China

## Abstract

Abiotic stresses comprise all nonliving factors, such as soil salinity, drought, extreme temperatures, and metal toxicity, posing a serious threat to agriculture and affecting the plant production around the world. Peanut (*Arachis hypogaea* L.) is one of the most important crops for vegetable oil, proteins, minerals, and vitamins in the world. Therefore, it is of importance to understand the molecular mechanism of peanut against salt stress. Six transcriptome sequencing libraries including 24-hour salt treatments and control samples were constructed from the young leaves of peanut. A comprehensive analysis between two groups detected 3,425 differentially expressed genes (DEGs) including 2,013 upregulated genes and 1,412 downregulated genes. Of these DEGs, 141 transcription factors (TFs) mainly consisting of MYB, AP2/ERF, WRKY, bHLH, and HSF were identified in response to salinity stress. Further, GO categories of the DEGs highly related to regulation of cell growth, cell periphery, sustained external encapsulating structure, cell wall organization or biogenesis, antioxidant activity, and peroxidase activity were significantly enriched for upregulated DEGs. The function of downregulated DEGs was mainly enriched in regulation of metabolic processes, oxidoreductase activity, and catalytic activity. Fourteen DEGs with response to salt tolerance were validated by real-time PCR. Taken together, the identification of DEGs' response to salt tolerance of cultivated peanut will provide a solid foundation for improving salt-tolerant peanut genetic manipulation in the future.

## 1. Introduction

Abiotic stresses comprise all nonliving factors, such as soil salinity, drought, extreme temperatures, and metal toxicity, giving rise to a serious threat to agriculture and affecting the plant production around the world [1]. With the increasing problems of global changes of climate, serious desertification of arable land, heavy metal population of soil, severe shortage of fresh water, and rapid growth of the population, it is very important to develop stress-resistant crops for sustaining growth and productivity in the abiotic stress conditions [2]. Soil salinity is one of the most destructive environment problems that can cause remarkable decrease of cultivated land area and crop productivity [3, 4] As the FAO (Food and Agriculture Organization) Land and Plant Nutrition Management Service reported, more than 6% of the Earth's lands are affected by salt. About 45-million-hectare irrigated lands, which accounts for 19.5% of 230 million hectares, are affected by salinity. Over 1500 million hectares are under dry land agriculture, while 32 million (2.1%) are salt-affected to varying degrees [5].

Soil salinity has two main effects on the plant growth either by forming an osmotic potential or by the ionic toxicity effects of Na^+^ and Cl^−^ ions on plant cells [6]. When salt stress happens, the high concentration of salt lowers the osmotic pressure; thus, the plant cannot take enough water as in the soil condition; the concentration is higher than that in the plant cells [7]. The result of salt stress is that the plant stomata will be closed to conserve water and will stimulate the production of reactive oxygen species (ROS) like hydrogen peroxide and superoxide anion in cells. The ROS further disturbs a series of the plant cell processes by causing damage to lipids, proteins, and nucleic acids [8]. Ionic toxicity is associated with the balance concentration of Na^+^/K^+^ and Na^+^/Ca^+^ ratio as accumulation of Na^+^ and Cl^−^ ions and can inhibit cellular metabolism processes by inhibiting the relative enzymatic activities in the cells [9, 10]. Na^+^ is the crucial ion toxicity in most of the saline soils. It can facilitate entry into or exit out of plant cells by several ion transporters. One of the key responses to plant salinity is to regulate and balance the cellular ion homeostasis via restricting the cumulation of the toxic Na^+^ [11]. Intracellular Na^+^ can be transported out of plant cells by SOS1 transporter via activation of the Salt Overly Sensitive (SOS) signaling pathway, or into the root xylem via the high-affinity potassium transporters (HKTs) [12–14]. Intracellular Na^+^ can also be isolated into vacuole by Na^+^/H^+^ antiporters localized in the tonoplast membrane [15].

Peanut (*Arachis hypogaea* L.), which is a Leguminosae family plant that is cultivated in semiarid tropical and subtropical regions, is one of the most economically important crops for vegetable oil, proteins, minerals, and vitamins in the world [16]. The cultivated peanut variety is originated from the two diploid wild progenitor species of *Arachis ipaensis* and *Arachis duranensis*. As a settled plant, peanut is unable to escape from abiotic stresses. Soil salinity stress is a major threat to peanut productivity [17, 18]. Plants can tolerate salinity stresses through modulating numerous genes and by coordinating the function of multiple genes from different metabolic pathways or regulatory systems [19]. Many studies have been conducted on physiological and biochemical changes in cultivated peanut under salt stress, but little information regarding genome and complete transcriptome of cultivated peanut is reported, and various studies about molecular mechanisms against salt stress focus on a few independent genes; therefore, it is difficult to obtain systematic biological genetic information about peanut against salt stress [18, 20–24].

Here, the next-generation transcriptome sequencing technique was performed to investigate the molecular foundation of wild peanut salinity tolerance. The high-throughput RNA sequencing as a revolutionary tool has been widely used to accurately monitor and quantify gene expression differences across the transcriptome under different treatments. In this study, a paired-end RNA sequencing was performed to examine the differential gene expression of cultivated peanut variety under long-term salinity stress conditions, based on our group's previous screening work [25] and the release of cultivated variety reference genome sequence by the International Peanut Genome Initiative (IPGI) group (https://www.peanutbase.org/peanut_genome). Several salt resistance genes were selected and validated using quantitative real-time reverse transcription polymerase chain reaction (qRT-PCR). The identification of the genes related to salt tolerance in cultivated peanut will provide a better understanding and cognition of the tolerance mechanism and further provide a wide gene resource to improve salt tolerance in the future peanut genetic manipulation.

## 2. Materials and Methods

### 2.1. Plant Material

The seeds of the drought-resistant cultivar Fenghua3 were incubated in pots with a mixture of vermiculite, perlite, and soil (1:1:1) at 26°C with photoperiod of 16 h. Seedlings were then grown in an artificial climate incubator for 14 days under restrained conditions (16 h light 26°C/8 h dark 22°C cycles). At the three-leaf stage, the robust overground parts and more uniform growing seedlings were harvested as control. The parts of these plants were then grown in salinity stress conditions with Hoagland's solution and 200 mM (1%) NaCl. The robust growing seedlings were harvested at 6, 12, 18, 24, and 48 hours later. Young leaves were immediately frozen with liquid nitrogen and stored at −80°C for further experiment. Three replicates were used for each time point.

### 2.2. Physiological Measurements

The catalase (CAT), peroxidase (POD), and superoxide dismutase (SOD) activities and malondialdehyde (MDA) content were measured in each sample under salinity stress using physiological assay kits (Nanjing Jiancheng Bioengineering Institute, Nanjing, China) and a UV-Vis spectrophotometer (Shimadzu, Kyoto, Japan) following the manufacturers' instructions. Three replicates were used for each time point of each sample. Statistical calculations were performed using SPSS 12.0 (SPSS Inc., Chicago, IL, USA) with the level of significance setting at *P* value ≤ 0.05.

### 2.3. RNA Sequencing

Total RNA was extracted from 20 mg young leaves using GeneJET Plant RNA Purification Mini Kit (Thermo Fisher Scientific, Waltham, Massachusetts, USA), and quantified by NanoDrop ND-2000 (Thermo Fisher Scientific, Waltham, Massachusetts, USA). RNA integrity was assessed using Agilent Bioanalyzer 2100 (Agilent Technologies, Santa Clara, California, USA). The samples with integrity number greater than 8 was used for library construction. The library of each sample was constructed using the TruSeq RNA Sample Preparation Kit (Illumina, San Diego, CA, USA) and SuperScript II Reverse Transcriptase (Invitrogen, Carlsbad, CA, USA). Paired-end sequencing was performed using an Illumina sequencing HiSeq 4000 platform with PE-150 module (Illumina, San Diego, CA, USA). The quality of all raw reads was firstly assessed by FastQC version 0.11.7 with default parameters (http://www.bioinformatics.babraham.ac.uk/projects/fastqc/). Adaptors and low-quality bases were trimmed using Trimmomatic version 0.38 with default parameters [26]. Reads with a Phred quality score above 30 were used for the following transcriptome analysis.

### 2.4. Transcript Assembly and Annotation

The peanut genomic sequences and gene annotation were downloaded from PeanutBase (https://www.peanutbase.org/peanut_genome). The clean reads from all six samples were mapped to peanut genome assembly using HISAT2 version 2.1.0 in strand-specific mode and other parameters with default settings [27]. The transcripts were assembled using StringTie version 1.3.4d with default parameters [28]. The abundance of all genes was determined using the “scaledTPM” method in txImport version 1.8.0 package with default parameters [29].

### 2.5. Differential Gene Expression Profiling

Differential gene expression analysis was performed using DEGseq2 version 1.22.2 [30] in a Bioconductor package. The false discovery rate (FDR) and log2FC (log of fold change) was calculated for all expressed genes, and only the genes with the absolute log2FC ≥ 2 and FDR ≤ 0.01 were considered differentially expressed between two groups. GO and KEGG functional enrichment analyses were conducted using the topGO package version 2.3.4 (http://bioconductor.org/packages/topGO/) with default parameters in a Bioconductor package and KOBAS version 3.0 with default parameters [31], respectively.

### 2.6. Real-Time Quantitative qPCR (qRT-PCR) Validation

Fourteen DEGs with a response to salinity stress were chosen for experimental validation by qRT-PCR. The primers of these genes were designed using the Beacon Designer 7.0 software.

Total RNA was isolated using the same method as mentioned in RNA sequencing. Single-stranded cDNAs were reverse-transcribed using PrimeScript RT reagent kit with gDNA Eraser (Perfect Real Time) (TaKaRa, Otsu, Shiga, Japan). The SYBR Premix Ex Taq™ TM II reagent with SYBR Green I was used for qRT-PCR analysis by ABI 7500 Fast Real-Time PCR System (Thermo Fisher Scientific, Waltham, Massachusetts, USA). Each reaction comprised 10 *μ*L 2X SYBR Premix Ex Taq II, 2 *μ*L of single-stranded cDNAs, and 0.4 *μ*M of forward and reverse primers in a final volume of 20 *μ*L, upon initial denaturation at 95°C for 30 s, 40 cycles of denaturation at 95°C for 5 s and 30 s of annealing at 60°C, and 10 s extension steps at 72°C. qRT-PCR assays were with three biological and technical replicates. The relative gene expression levels were measured using the 2^–*ΔΔ*CT^ method [32] and normalized against the amount of the Actin gene [33]. All specific primers are listed in [Supplementary-material supplementary-material-1].

## 3. Results

### 3.1. Physiological Characteristics

**Figure 1:**
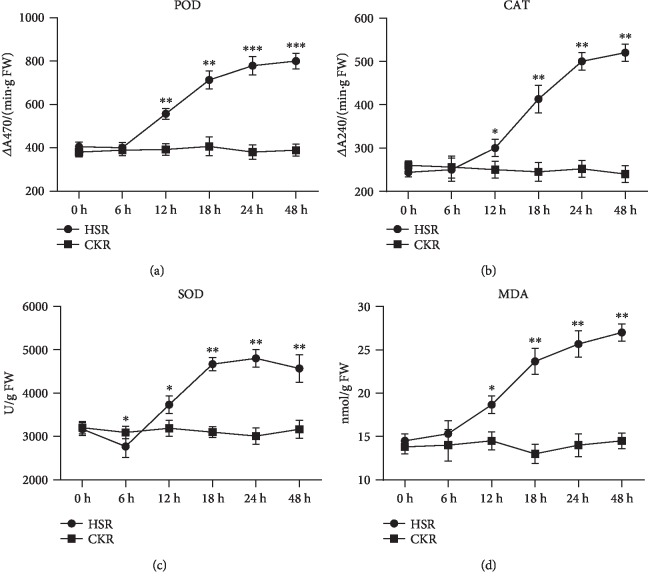
The physiological characteristic of peanut under salt tolerance conditions: (a) POD activity, (b) CAT activity, (c) SOD activity, and (d) MDA content. The *x*-axis represents the treatment time (h). The *y*-axis represents the physiological characteristics. All units are U/mg prot. The data was collected from three independent experiments. Vertical bars represent standard deviation. The denotation of the samples is listed on the top-left corner. The figure was created by GraphPad Prism version 8.0.

The value of POD activity in leaf tissues showed no significance for the first 6 hours and then exhibited a marked increase by 137%, 176%, 192%, and 197% under 12 h, 18 h, 24 h, and 48 h with salinity stress, respectively, compared with the control (*P* ≤ 0.01) (Figure 1(a)). CAT activity was observed to have the same tendency with POD. Furthermore, CAT activity had a significant increase by 123%, 169%, 205%, and 213% under 12 h, 18 h, 24 h and 48 h with salt treated, respectively, as compared with the control (*P* ≤ 0.05) (Figure 1(b)). As compared with the control, results showed that SOD activity had a significant decrease by 144% in the first 6 h (*P* ≤ 0.05) and then was significantly enhanced by 118%, 147%, 152%, and 144% under 12 h, 18 h, 24 h, and 48 h with salinity stress, respectively (*P* ≤ 0.01) (Figure 1(c)). The oxidative degradation was recognized as malondialdehyde (MDA) content, which is the product of lipid peroxidation. The results showed that in all the time points, lipid peroxidation was significantly affected by salinity stress (*P* ≤ 0.05). As compared with the control, salinity levels at 200 mM caused 129%, 163%, 177%, and 186% increase in 12 h, 18 h, 24 h, and 48 h, respectively (Figure 1(d)). In conclusion, the results of physiological characteristics revealed that the genes related to salinity stress were changed after 6 h salt treatment, which is consistent with our group's previous report [25]. The young leaves of peanut seedlings growing within salinity stress conditions for 24 h were used to perform transcriptome analysis as the curves of physiological traits reached their peaks after salt stress.

### 3.2. Transcriptome Sequencing and Assembly

To obtain the global scenario of peanut gene expression under salinity stress, six cDNA libraries (three replicates for each group) were performed for Illumina RNA sequencing. After adaptor removal and quality filtering, a total of 163.33 million clean reads were obtained with a Q30 ratio more than 90.63% (Table 1). The quality-checked high-quality sequencing reads of each sample were individually aligned to peanut genome assembly using HISAT2. Of the total reads, a range of 76.7%-77.30% clean reads were mapped to peanut genome assembly (Table 1). After read aligning and assembling, 53,653 genes were identified in the transcriptome and used to perform analysis of the differentially expressed genes.

### 3.3. Identification of DEGs Involved in Salinity Stress

**Figure 2:**
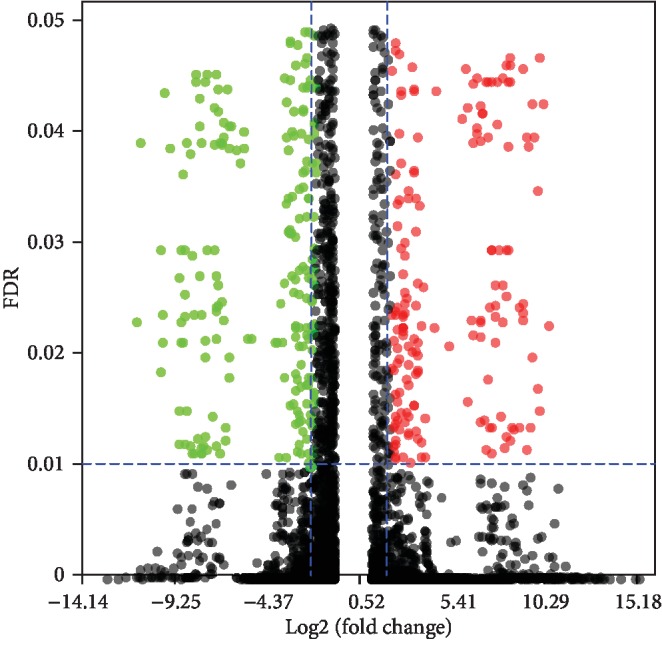
Volcano plot. The *x*-axis represents log of fold change. The *y*-axis represents FDR. Red dot indicates upregulated DEGs with FDR ≤ 0.01 and log FC ≥ 2. Green dot indicates downregulated DEGs with FDR ≤ 0.01 and log FC≤−2. Black dot represents not significant genes out of the filter criteria.

Based on gene expression level, differential gene expression analysis (see Differential Gene Expression Profiling for details) was implemented between salt-treated (HSR) and the control (CKR) samples. The result showed that in salinity stress, a total of 3,425 genes were found to be differentially expressed with FDR ≤ 0.01 and absolute log FC ≥ 2, of which comprised 2,013 upregulated genes and 1,412 downregulated genes (Figure 2, [Supplementary-material supplementary-material-1]). Among the DEGs, 141 transcription factors (TFs) (89 upregulated and 52 downregulated) were identified in response to salinity stress, which were assigned to ten TF families ([Supplementary-material supplementary-material-1]). The TF families of MYB, AP2/ERF, WRKY, bHLH, and HSF were included with relatively largest volumes representing 87.94% of all TFs (Table 2).

### 3.4. Functional Enrichment Analysis of DEGs to Salinity Stress

**Figure 3:**
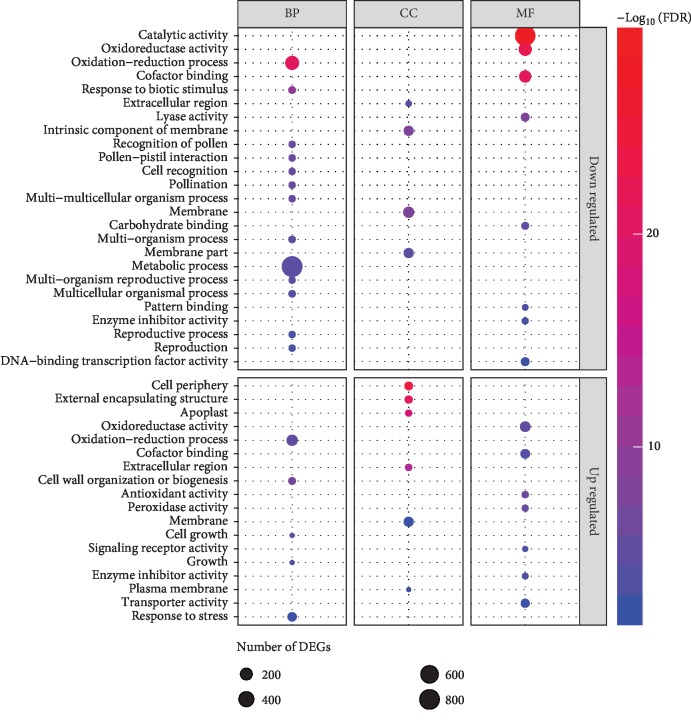
GO enrichment analysis of up- and downregulated DEGs. The node size represents the gene number enriched in each GO category. Color bar illuminates FDR from red (low) to blue (high).

To determine the GO functions of DEGs involved in salinity stress, topGO tool was used for enrichment analysis of GO terms in the whole transcriptome analysis. The GO functional enrichment analysis showed that the upregulated and downregulated DEGs were significantly enriched in different GO categories ([Supplementary-material supplementary-material-1]). For the upregulated DEGs, 18 GO categories were significantly enriched at term level of 3 with FDR ≤ 0.01 (Figure 3, [Supplementary-material supplementary-material-1]). These GO terms were highly related to regulation of extensive biological activities, such as oxidation-reduction process (FDR = 1.10*E*-05), response to stress (FDR = 3.92*E*-03), oxidoreductase activity (FDR = 6.40*E*-06), antioxidant activity (FDR = 1.80*E*-06), peroxidase activity (FDR = 3.60*E*-06), enzyme inhibitor activity (2.20*E*-04), transporter activity (FDR = 3.20*E*-03), and cell periphery (FDR = 9.60*E*-25). GO function enrichment analysis of downregulated DEGs showed that the GO terms of oxidation-reduction process (FDR = 1.1*E*-21), response to biotic stimulus (FDR = 1.00*E*-10), metabolic process (FDR = 2.50*E*-05), catalytic activity (FDR = 1.00*E*-29), oxidoreductase activity (FDR = 2.40*E*-23), and oxidoreductase activity (FDR = 2.40*E*-23) were mostly enriched (Figure 3, [Supplementary-material supplementary-material-1]). In comparison with the functional enrichment of upregulated DEGs, the downregulated DEGs were mainly involved in regulation of metabolic process, oxidoreductase activity, and catalytic activity. Meanwhile, the upregulated genes in the salinity stress were involved mostly in regulation of cell growth and cell periphery, sustained external encapsulating structure, cell wall organization or biogenesis, antioxidant activity, and peroxidase activity (Figure 3, [Supplementary-material supplementary-material-1]), which was consistent with our physiological characteristics.

**Figure 4:**
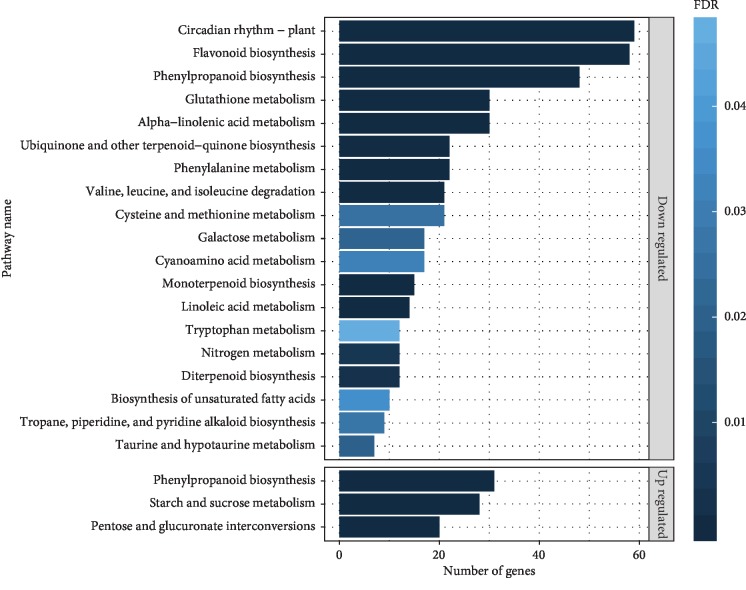
KEGG pathway enrichment analysis of up and downregulated DEGs. The *x*-axis represents the number of enriched DEGs. The *y*-axis represents the KEGG pathway. Color bar illuminates FDR from dark blue (low) to light blue (high).

To understand the gene functions and pathways, the DEGs were analyzed using KOBAS tool with KEGG database. The results showed that three pathways were significantly enriched in upregulated DEGs and 19 pathways enriched for downregulated DEGs. Of these, the pathways of phenylpropanoid biosynthesis (FDR = 1.72*E*-10), pentose and glucuronate interconversions (FDR = 1.00*E*-08), and Starch and sucrose metabolism (FDR = 1.43*E*-07) were most significant in upregulated DEGs (Figure 4, [Supplementary-material supplementary-material-1]). For downregulated genes, a comparatively large number of pathways were significantly enriched. The pathways of flavonoid biosynthesis (FDR = 7.27*E*-34), circadian rhythm of plant (FDR = 7.37*E*-29), alpha-linolenic acid metabolism (FDR = 2.69*E*-09), phenylpropanoid biosynthesis (FDR = 2.31*E*-06), and glutathione metabolism (FDR = 1.79*E*-05) were dramatically enriched with at least 30 DEGs involved (Figure 4, [Supplementary-material supplementary-material-1]). KEGG enrichment analysis indicated that the DEGs involved in those pathways were possibly linked to salt tolerance.

### 3.5. qRT-PCR Validation

**Figure 5:**
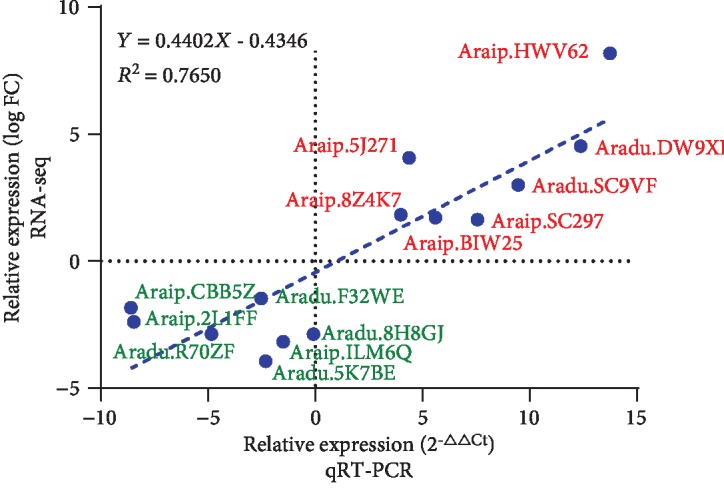
qRT-PCR validation of fourteen DEGs. All experiments were performed in triplicate. The *y*-axis represents the log FC from RNA-seq. The *x*-axis represents the relative expression level from qRT-PCR. The correlation coefficient (*R*^2^) is indicated in the figure. The figure was created by GraphPad Prism version 8.0.

To validate the expression profile, fourteen DEGs with response to salt tolerance, involved in plant hormone signaling, transcription factors, secondary metabolism, and oxidative damage, were chosen for experimental validation by qRT-PCR ([Supplementary-material supplementary-material-1]). As shown in Figure 5, the results indicated that the gene expression changes analyzed by qRT-PCR were mostly consistent with those obtained by Illumina RNA sequencing except for Aradu.8H8GJ, representing 92.86% consistency.

## 4. Discussion

Transcriptomic approach is an effective tool which provides a global information towards the gene expression patterns of a plant organism under any conditions, such as drought, light, temperature, aflatoxin, and salinity stress. Numerous studies about transcriptomic characteristics of peanut have been conducted to evaluate the expression pattern of inducible genes under abiotic and biotic stress conditions [25, 34–39]. Transcriptome data are valuable resources for exploring plants under stress conditions. To our knowledge, there have been an increasing number of studies on transcriptome analysis of legume species using high-throughput RNA sequencing approach, among those regarding the effects of salinity stress were reported on Medicago [40–42], glycine [43–45], and common bean [46–48]. Studies focusing on transcriptome research of the peanut under salt stress are rare, but there exists a substantial amount of reports regarding the physiological responses to salt stress in peanut based on a *de novo* transcriptomic sequence assembly method [49–51]. In this study, a high-throughput RNA sequencing of cultivated peanut variety under long-term salinity stress conditions was performed and deeply analyzed, which will contribute to a well annotation of cultivated peanut reference genome by the IPGI group. Furthermore, GO and KEGG functional enrichment analyses were used to better understand the functions of DEGs in salt tolerance conditions. The DEGs were mainly involved in oxidation-reduction process; oxidoreductase activity; cell wall organization or biogenesis; response to stress; cofactor binding; metabolic process, such as starch and sucrose metabolism; circadian rhythm; flavonoid biosynthesis; and nitrogen metabolism, which showed significance in responds to salinity stress (Figures 3 and 4).

**Figure 6:**
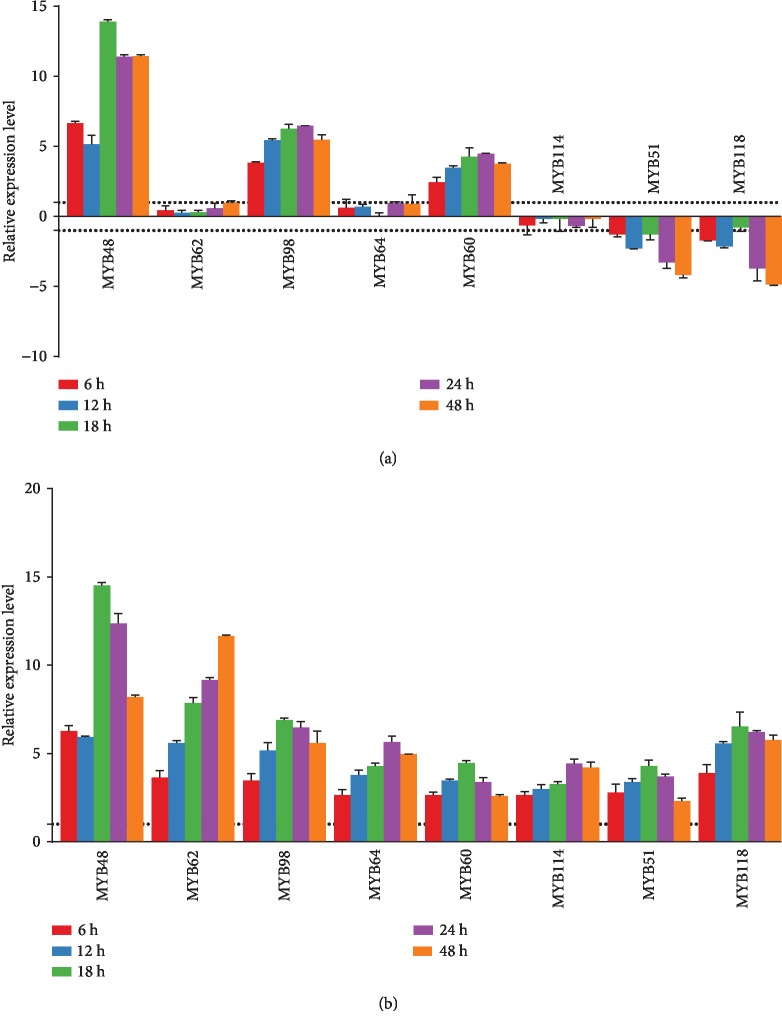
qRT-PCR validation of eight MYB genes. (a) qRT-PCR validation in leaf tissues. (b) qRT-PCR validation in root tissues. All experiments were performed in triplicate. The denotation of the samples is listed on the top-right corner. The *y*-axis represents the relative expression level of DEGs. Vertical bars represent standard deviation. The figure was created by GraphPad Prism version 8.0.

Salt tolerance is a complicated character that is controlled by multiple different genes especially TFs in plants [52]. During the response to salt tolerance stress, a number of genes were activated, leading to the accumulation of numerous proteins involved in resistance to abiotic stress, which were mostly regulated by specific TFs [53]. Among these TFs, the MYB family has been well characterized for their regulatory roles in the response of plants to abiotic stress [54, 55]. Several MYB genes have been identified as key factors in the signaling pathways for *Arabidopsis* and rice resistance to abiotic stresses [56, 57]. MYB family proteins were also demonstrated in plants as a regulator for mediating salt tolerance gene expression under abiotic stress [58, 59]. A previous study reported that the MYB family proteins were the second most highly expressed TF family in drought tolerance *Arachis* plants [34]. In our study, the TF expression pattern analysis indicated that MYB family genes responding to salt stress were in large quantities including 47 DEGs. Eight DEGs involved in the MYB family (*MYB48*, *MYB51*, *MYB60*, *MYB62*, *MYB64*, *MYB98*, *MYB114*, and *MYB118*) were further selected and validated by qRT-PCR with root and leaf tissues among all time points under salt tolerance. The result showed that *MYB48*, *MYB60*, and *MYB98* were consistently increased, and the expressions of *MYB51* and *MYB118* were decreased among time points in leaf tissues under additional salt (Figure 6(a)). As compared with the gene expression patterns in leaf tissues, all MYB genes exhibited a consistent increase among all time points with salt tolerance (Figure 6(b)). The observed is not difficult to understand as the root is the one initially exposed to salt stress. Among these TFs, one (MYB118) encodes R2R3 MYB proteins and others encode the MYB-related proteins. In contrast to the R2R3-MYB genes, the MYB-related genes have attracted little attention, and few have been studied functionally. Previous studies found that CCA1-like and CPC-like genes could involve in the maintenance of circadian rhythms [60–62] and in control of cellular morphogenesis [63]. A novel potato single MYB-like domain protein (StMYB1R-1) played positive roles in potato drought resistance, according to Shin et al. [64]. Our research provides evidence that the MYB-related proteins may be involved in peanut salinity stress regulation and needs more investigation in the future work.

Salt tolerance significantly affected the core genes related to a series of secondary metabolism, such as flavonoid biosynthesis, phenylpropanoid biosynthesis, starch and sucrose metabolism, circadian rhythm, and nitrogen metabolism. Previous studies reported that the flavonoid pathway was mostly induced with response to a series environmental stress as a protective mechanism to oxidative stresses induced by metal ions, high light, temperature, drought, salt, or C nutrition in plants [65–68]. Many studies revealed that with plant under drought conditions, the expression levels of two key enzymes participating in flavonoid biosynthesis were always increased [69, 70]. In our study, the expression levels of two key enzymes, chalcone synthase (51 DEGs) and chalcone-flavanone isomerase family protein (one DEG) involved in flavonoid biosynthesis, were also observed to significantly increase with salt stress conditions. As the precursors of a wide range of phenolic compounds, phenylpropanoid compounds were explored to have the important roles in a number of plant protection mechanisms such as plant defense response, abiotic stresses, and signal transduction [71, 72]. PAL (phenylalanine ammonia lyase), regarded as a central enzyme in the phenylpropanoid pathway to catalyze the deamination of phenylalanine to provide cinnamic acid, was investigated in *Salvia* species, and the result showed that salt stress could increase PAL activity and total phenolic accumulation in the early few hours with stress treatment [73]. Our result had a consistence with these findings and showed that four PAL genes (*Aradu.IU1HH*, *Araip.69J63*, *Araip.GM19P*, and *Araip.V9S7Z*) were increased with at least 4-folds ([Supplementary-material supplementary-material-1]). Nitrogen is an essential nutrient for plant growth, development, and reproduction. As a major source of nitrogen provider of plants, nitrate assimilation was specifically affected by salinity with subsequently inactivation of the nitrate reductase [74]. In our study, the expression levels of 11 nitrate transporter coding genes were decreased under salt stress. This indicated that nitrogen metabolism in peanut with salinity stress was decreased. Glutathione transferases (GSTs) are induced with a series of biotic and abiotic stresses in plant, which are important for protecting host plants against oxidative damage [75]. About 25 glutathione S-transferase family coding genes showed differential expression increasing patterns when exposed to salt stress in our data ([Supplementary-material supplementary-material-1]), which indicated that the GSTs in peanut could enhance salt tolerance. Moreover, in our study, the most of POD-encoding genes showed significantly decrease under salt stress; also, the all CAT- and SOD-encoding genes showed slight decrease with salt stress ([Supplementary-material supplementary-material-1]), which were inconsistent with our physiological characteristics. This phenomenon may be caused by several complex biological processes and posttranscriptional regulation by noncoding RNAs and still need to be further studied.

Multiple membrane ion transporters and pH-related transporters were proposed to mediate plant cellular signaling under salinity stress [11, 13]. The preset studies indicated that the *Arabidopsis* transporter HKT functioned as a salt tolerance determinant and mediates Na^+^ influx and K^+^ transport in plants [14, 76]. Research in *Arabidopsis* revealed that reduction of the *AtHKT1* gene expression could lead to hypersensitivity to salt ion with more Na^+^ accumulated in the leaves while directly stimulating K^+^ loading into the xylem by AtHKT [77]. In our findings, the expression level of *Araip.58313* (high-affinity K^+^ transporter 1, homologous *Arabidopsis* HKT1) was found significantly decreased under salt stress conditions ([Supplementary-material supplementary-material-1]). Our result may indicate that the peanut growing in salinity stress could reduce the Na^+^/K^+^ ratio in the leaves via reducing the inhibition of K^+^ uptake. The SOS signaling pathway comprising SOS3, SOS2, and SOS1 has been recognized as key mechanism to maintain ion homeostasis by controlling the cellular signaling under salinity stress [78]. In our study, the expression level of *Araip.TT7Q9* (homologous *Arabidopsis* SOS1), *Aradu.FWA9B* (homologous *Arabidopsis* SOS2), and *Araip.IZ8TF* (homologous *Arabidopsis* SOS3) was observed to be significantly changed by comparing with control ([Supplementary-material supplementary-material-1]), which indicated that salinity stress may activate the Na^+^/H^+^ antiporter and facilitate Na^+^ efflux through cellular plasma membrane to regulate vacuolar H^+^-ATPase activity. The expression and activity of vacuolar H^+^-ATPase (V-ATPase) or vacuolar pyrophosphatase (V-PPase) facilitated the Na^+^ sequestration into vacuoles [79]. In our findings, the expression levels of three V-ATPase coding genes (*Aradu.0U1AX*, *Aradu.ZRX72*, and *Araip.08L1N*) and two V-PPase coding genes (*Aradu.PJ77P* and *Araip.CC90S*) were also significantly changed with salinity stress conditions ([Supplementary-material supplementary-material-1]). These results indicated that V-ATPase and V-PPase in peanut may enhance the Na^+^ sequestration into vacuoles and modulate ion homeostasis in high salinity stress conditions.

In summary, we performed the transcriptome analysis of a cultivated peanut variety under long-term salinity stress conditions based on a well annotation of cultivated peanut reference genome. Functional analysis of DEGs showed that salt tolerance genes were mainly involved in oxidation-reduction process; oxidoreductase activity; cell wall organization or biogenesis; response to stress; cofactor binding; metabolic process, such as starch and sucrose metabolism; circadian rhythm; flavonoid biosynthesis; and nitrogen metabolism, which showed significant in response to salinity stress. The identification of differentially expressed salt tolerance genes in cultivated peanut will provide a better understanding and cognition of the tolerance mechanism and will further provide reference for improving salt-tolerant peanut genetic manipulation in the future.

## Figures and Tables

**Table 1:** Statistics of transcriptome sequencing data.

Sample	Number of reads	Number of bases	GC percentage	Q20	Q30	Genome mapping ratio
CKR1	23,635,337	7,090,601,100	45.15%	95.85%	90.76%	77.30%
CKR2	26,509,428	7,952,828,400	44.81%	96.16%	91.11%	76.90%
CKR3	26,246,422	7,873,926,600	44.66%	96.27%	91.36%	76.70%
HSR1	24,238,775	7,271,632,500	44.77%	96.19%	91.18%	76.90%
HSR2	31,037,081	9,311,124,300	44.69%	96.24%	91.33%	76.90%
HSR3	31,658,041	9,497,412,300	44.75%	95.76%	90.63%	76.80%
Total	163,325,084	48,997,525,200	—	—	—	—

**Table 2:** Summary of the differentially expressed TFs.

TF family	Gene ID	Number
MYB	Aradu.X7LBF, Araip.2H669, Aradu.59AEA, Araip.273K6, Aradu.L6QML, Aradu.N4Y9K, Aradu.X0QAS, Araip.N0GNU, Araip.W7HYQ, Araip.D9D8Y, Aradu.G26I2, Araip.PUS3S, Aradu.62DXS, Aradu.9DD9N, Aradu.1DV6R, Araip.76FEY, Aradu.P9RPB, Aradu.G7UXI, Aradu.NSL0R, Araip.MJ3JY, Aradu.0Z2ZN, Araip.6K0VA, Aradu.CM6S6, Araip.P53WL, Aradu.CT448, Araip.VH6HT, Aradu.20INS, Araip.U6RZ5, Araip.M7SF9, Aradu.D0JN5, Aradu.DW9XI, Aradu.K3TU7, Aradu.K8V1Y, Araip.0AG3E, Araip.Q811U, Araip.TSY02, Aradu.FER0N, Aradu.IC1L6, Araip.45253, Aradu.C1QBJ, Araip.6FJ5I, Araip.XK813, Araip.ZG61K, Araip.0FI9Y, Aradu.0BT33, Araip.B91MN, Araip.ZD4VN	47
AP2/ERF	Araip.VY42Y, Aradu.LT83G, Aradu.H9U8I, Aradu.JL25Y, Aradu.ME4LN, Aradu.NK24P, Araip.3W5AL, Araip.88WSL, Araip.KL8NJ, Aradu.I9TTZ, Aradu.K41I0, Araip.62IDN, Aradu.3R22E, Aradu.B90GQ, Araip.BUP6F, Araip.T3D3V, Araip.QQN2T, Aradu.UA79E, Araip.E0UEG, Araip.0BU95, Araip.Z2VYZ, Aradu.GB4U4, Araip.3JJ8N, Araip.KAK82, Aradu.UTD2W, Araip.DLE1Q, Araip.P3BCC, Araip.8B6ML, Araip.QDN6F	29
WRKY	Aradu.V6U4I, Araip.1G97J, Araip.R5S93, Aradu.171F6, Aradu.B1C6F, Araip.D0ST6, Aradu.I8GKQ, Araip.761TD, Araip.B07N1, Araip.N4R5C, Aradu.RZ9Z0, Araip.HGN3R, Araip.X9JQZ, Aradu.1C3SG, Aradu.7AC9I, Aradu.M1GCR, Aradu.VL3IZ, Araip.15EJY, Araip.AR7FU, Aradu.180Q6, Araip.LH5AE, Araip.LSC0K	22
bHLH	Araip.CX0J5, Aradu.JT48R, Araip.L83F8, Aradu.T39L1, Araip.74SLF, Aradu.T3S5X, Araip.MY816, Aradu.K1HGS, Aradu.D29PK, Aradu.JI62L, Aradu.R70ZF, Araip.93FBI, Araip.YT7PR, Araip.LP6IV, Araip.DYV42, Aradu.01B4C, Araip.4C1AU, Aradu.WC9V5, Araip.JIB5P	19
HSF	Aradu.MA5WH, Aradu.U9IPR, Araip.24AK5, Aradu.E740H, Aradu.X3DNX, Araip.UGV6F, Araip.FU4GL	7
MADS-box	Araip.D4LH7, Aradu.TB6GC, Araip.EAZ0R, Aradu.QT7J0, Araip.33EMF	5
bZIP	Aradu.YM0TI, Aradu.54E1H, Araip.LXV0U	3
Other	Aradu.53P8J, Araip.2A2GH, Aradu.RYS1S	3
E2F	Aradu.HB8K6, Araip.HB9YP	2
Trihelix	Aradu.B6WSD, Araip.A9IXU	2
GLABRA 3-like	Aradu.QW16A	1
RADIALIS-like	Aradu.R0TBT	1

## Data Availability

The RNA sequencing data used to support the findings of this study have been deposited in the Short Read Archive at NCBI with accession number of SRR8177741. The qRT-PCR validated genes were submitted to NCBI with GeneBank accession number as following: Aradu.SC9VF: MK956111, Aradu.R70ZF: MK956113, Aradu.8H8GJ: MK956114, Aradu.F32WE: MK956115, Aradu.5K7BE: MK956116, Araip.HWV62: MK956121, Araip.SC297: MK956122, Araip.8Z4K7: MK956123, Araip.BIW25: MK956124, Araip.5J271: MK956125, Araip.CBB5Z: MK956126, Araip.2L1FF: MK956127, Araip.ILM6Q: MK956128, Araip.84L6B: MK956129, MYB64: MK956130, MYB114: MK956131, MYB118: MK956132, MYB62: MK956117, MYB98: MK956118, MYB60: MK956119, MYB48: MK956112, MYB51: MK956120.

## Supplementary Materials

Supplementary 1 Supplementary Table S1: the DEGs identified in response to salt tolerance.

Supplementary 2 Supplementary Table S2: the differentially expressed TFs identified in response to salt tolerance.

Supplementary 3 Supplementary Table S3: the GO enrichment analysis of DEGs.

Supplementary 4 Supplementary Table S4: the KEGG pathway enrichment analysis of DEGs.

Supplementary 5 Supplementary Table S5: the qRT-PCR primers of selected genes.
